# Widely targeted metabolomics analysis reveals the formation of nonvolatile flavor qualities during oolong tea manufacturing: a case study of Jinguanyin

**DOI:** 10.3389/fnut.2023.1283960

**Published:** 2023-12-14

**Authors:** Qingcai Hu, Yucheng Zheng, Yun Yang, Zi-Xin Ni, Bin Chen, Zongjie Wu, Huiqing Huang, Qingyang Wu, Zi-wei Zhou, Shuilian Gao, Zhongxiong Lai, Hongzheng Lin, Yun Sun

**Affiliations:** ^1^Key Laboratory of Tea Science, College of Horticulture, Fujian Agriculture and Forestry University, Fuzhou, China; ^2^Institute of Horticultural Biotechnology, Fujian Agriculture and Forestry University, Fuzhou, China; ^3^College of Tea and Food Science, Wuyi University, Nanping, China; ^4^College of Life Science, Ningde Normal University, Ningde, China; ^5^Anxi College of Tea Science, Fujian Agriculture and Forestry University, Quanzhou, China

**Keywords:** oolong tea, postharvest processing, nonvolatile components, tasting qualities, widely targeted metabolome, Jinguanyin

## Abstract

**Background:**

The manufacturing processes of oolong tea significantly impact its nonvolatile components, leading to the emergence of distinct flavor attributes. Understanding the dynamic changes in nonvolatile components during the manufacturing stages of the Jinguanyin (JGY) cultivar is crucial for unraveling the potential mechanism behind flavor formation.

**Methods:**

Comprehensive metabolomics and sensomics analyses were conducted to investigate the dynamic changes in nonvolatile components throughout various phases of oolong tea processing, focusing on the JGY cultivar.

**Results:**

A total of 1,005 nonvolatile metabolites were detected, with 562 recognized as significant differential metabolites during various phases of oolong tea processing. Notably, the third turning-over, third setting, and high-temperature treatments exhibited the most significant effects on the nonvolatile metabolites of oolong tea. JGY finished tea demonstrated a characteristic flavor profile, marked by mellowness, sweetness in aftertaste, and a significant Yin rhyme. This flavor profile was collectively promoted by the accumulation of amino acids and organic acids, the decrease in flavonols (3-O-glycosides) and sugar substances, the alteration of phenolic acids, and the stabilization of caffeine.

**Conclusion:**

This study contribute to the understanding of the formation of oolong tea flavor qualities. The dynamic changes observed in various types of nonvolatile compounds during oolong tea processing shed light on the intricate interplay of metabolites and their influence on the final flavor characteristics.

## 1 Introduction

Oolong tea, a semifermented tea, is favored by consumers for its unique floral characteristics and flavor qualities (1). In contrast to the floral characteristics, the flavor qualities (mellow, smooth taste) of oolong tea have yet to be explored. The distinctive flavor qualities of oolong tea are significantly related to the manufacturing process and tea varieties due to the variation in nonvolatile compounds in oolong tea products (2).

The manufacturing procedures of oolong tea are separated into two phases, namely, an enzymatic-catalyzed process (ECP) and a nonenzymatic-catalyzed process (NCP), and their dividing boundary is the ‘firing’ process, which enables cells of fresh leaves to become inactivated (3, 4). Recent research shows that leaf dehydration and mechanical wounding are crucial for forming the characteristic flavor of Zhangping Shuixian tea under sun-withering and turning-over treatments, and the sensory intensity of all manufactured samples was found to gradually decrease with the processing of oolong tea (5). Generally, the formation of the characteristic flavor is a consequence of multiple stresses in the postharvest manufacturing of oolong tea (6). While many studies have been centered on using different tea varieties to understand and investigate the characteristic flavor and formation mechanism in each manufacturing process of oolong tea, such as Tieguanyin after roasting (7), Shuixian tea after long-term storage (8), Wuyi rock tea produced with four tea cultivars (including Rougui, Dahongpao, Qizhong and Shuixian) at three roasting levels (low, moderate and sufficient) (9) and oolong tea processed with Chin-Hsin-Dah-Pang and Chin-Hsin-Gan-Tzu two cultivars at the turning-over stage (10). Meanwhile, nonvolatile compounds linked to tea taste have been categorized according to five tasting properties, namely, bitterness, astringency, umami, sweetness, and sourness (11), and the characteristic flavor of oolong tea has an important correlation with both their contents and types. However, the formation of characteristic flavor of oolong tea produced by the Jinguanyin cultivar has not been explored during manufacturing.

As a typical and elite tea cultivar, *Camellia sinensis* cv. Jinguanyin (JGY, No. GS2002017), is one of the offspring of the parent cultivars Tieguanyin and Huangdan, which showed nonadditive accumulation and various heterosis result in hybrids for some metabolites (12, 13). The JGY cultivar is broadly utilized for processing into tea products in the southern part of Fujian Province, and its oolong tea products have an sweet flower, fruit, and “Yin rhyme” fragrance (14, 15). While previous research focused on the fresh leaf and finished tea flavor qualities of JGY, the dynamic changes in nonvolatile compounds during its processing have not been investigated. Hence, we utilized widely targeted metabolomics analysis to study the nonvolatile components of JGY processing, which can provide an exhaustive and methodical understanding of flavor formation and further elucidate the influence of manufacturing processes on the characteristic quality metabolites of oolong tea.

## 2 Materials and methods

### 2.1 Plant materials

Tea shoots were harvested from the tea plant *Camellia sinensis* (L.) Jinguanyin to obtain disease-free samples with one bud and three leaves in April 2021. The tea plants were cultivated in an artificial tea plantation in Anxi County, Fujian Province (24.55°N, 117.50°E), China. JGY was extensively planted and processed into oolong tea because of its excellent qualities and high economic efficiency.

### 2.2 Tea manufacturing and sample preparation

**Figure 1:**
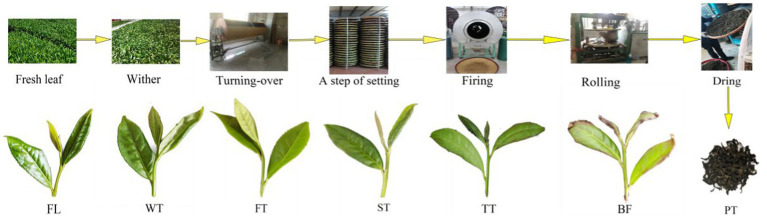
Oolong tea manufacturing processes and all sample points.

The tea was manufactured according to prior research with some modifications to achieve the flavor characteristics of Anxi Tieguanyin (4). The manufacturing processes of oolong tea and the sampling points used in this study are described as follows (Figure 1). The withering of fresh leaves (WT) was performed by placing them indoors for a three-hour resting period and flipping between them twice. Then, tea samples were turned over in a rotary machine, followed by a setting step of standing and spreading out on bamboo sieves, where the steps of turning-over and setting were performed three times. All setting steps were carried out in an air-conditioned room (temperature 20°C, relative humidity 60%), and the relevant parameters are presented in Table 1. Immediately thereafter, the tea samples were subjected to a firing treatment with a rotary drum dehydrating machine for 3 min at 270°C. Finally, the mixtures were rolled at room temperature (26°C–28°C) and dried at 65°C to yield the tea product (PT). The samples included fresh leaf (FL), after withering leaf (WT), after first turning-over leaf (FT), after second turning-over leaf (ST), after third turning-over leaf (TT), before firing leaf (BF), and tea product (PT). Three biological replicates for each sample point were obtained, immediately frozen and fixed in liquid nitrogen, and then stored in a −80°C refrigerator for study.

**Table 1:** Working times of various processes during the processing of oolong tea.

Sample point	Processing time	Sample ID
Fresh leaf (JGY)	—	FL
Withering	3 h	WT
The first turning-over	3 min	FT
The first setting/Tanqing	1.5 h	-
The second turning-over	4 min	ST
The second setting/Tanqing	3 h	—
The third turning-over	21 min	TT
The third setting/Tanqing (before firing)	11 h	BF
Tea product	—	PT

### 2.3 Electronic tongue and sensory evaluation of JGY tea product

Electronic tongue (TS-5000Z, Insent Electricity Company, Japan) was used to assess tasting attributes of finished tea, and this machine which has six basic taste sensors contributed to evaluation of various tasting attributes. JGY tea product (3.0 g) was brewed of 150 mL boiling water for 5 min, then rapidly filtered and cooled to indoor temperature. Finally, the filtered tea infusion was detected and repeated three times. Simultaneously, the mixed liquids of 30 mM potassium chloride and 0.3 mM tartaric acid were used as reference solution and as pre-treatment solution of taste sensors (tasteless solution).

JGY tea product was performed to sensory evaluation according to previous studies (15, 16), panelists (five females and five males, 20 to 50 years old) had more than 5 years of sensory evaluation experience and were trained. Brewing was conducted according to the review method with cylindrical cups in the methodology for sensory evaluation of tea (GB/T 23776-2018), 3.0 g finished tea was immersed for 5 min with boiling water. Then intensity (0–10) of tasting attributes (mellow, umami, astringency, bitterness, and thick) was recorded and scored, and different intensities of taste attributes represented by 0 to 10 were also used in previous studies (17).

### 2.4 Metabolite extraction and analysis

The metabolites of all samples in this experiment were extracted according to previous studies (18). Specifically, frozen samples were freeze-dried using a vacuum freeze-dryer (Scientz-100F) and crushed at 30 Hz for 1.5 min using a mixer mill (MM400, Retsch) with zirconia beads. One hundred milligrams of lyophilized powder was solubilized in 1.2 mL of 70% methanol solution, rotated every 30 min for 30 s six times, and left overnight in a refrigerator at 4°C. The samples were centrifuged at 12,000 rpm for 10 min and filtered prior to UPLC-MS/MS analysis (SCAA-104, 0.22 μm pore size; ANPEL, Shanghai, China, http://www.anpel.com.cn/). The quality control sample was a mixture of all materials (mix) in equally divided masses, which was used to ensure the stability and reproducibility of the experiment.

A UPLC-ESI-MS/MS system (UPLC, SHIMADZU NexeraX2, www.shimadzu.com.cn/; MS, Applied Biosystems 4,500 Q TRAP, www.appliedbiosystems.com.cn/) was used to analyze these example extracts. The analysis was conducted with a UPLC column (Agilent SB-C18, 1.8 μm, 2.1 mm × 100 mm). Mobile phases A and B were pure water with 0.1% formic acid and acetonitrile with 0.1% formic acid, respectively. Sample measurements were obtained using a gradient program that employed the starting conditions of 95% A and 5% B. A linear gradient was programmed to 5% A and 95% B over 9 min, and the 5% A and 95% B composition was maintained for 1 min. Afterward, a 95% A and 5.0% B composition was reached within 1.10 min and maintained for 2.9 min. The flow rate was 0.35 mL/min, the column oven temperature was 40°C, and the injection volume was 4 μL.

Eluate connection to a triple quadrupole-linear ion trap mass spectrometer (Q TRAP) in an AB4500 Q TRAP UPLC/MS/MS system with an ESI Turbo Ion-Spray interface, operating in positive and negative ion modes under the control of Analyst 1.6.3 software (AB Sciex), was used to perform LIT and triple quadrupole (QQQ) scanning, simultaneous instrument tuning and mass calibration with 10 and 100 μmol/L solutions of polypropylene diol. Qualitative and quantitative profiling of metabolites was conducted based on retention time (RT), fragment patterns, accurate m/z values and MWBD (home-made database, Metwwere, Wuhan, China) (18). Quantitation of metabolites was accomplished using triple quadrupole mass spectrometry in multiple reaction detection mode (MRM). Upon acquiring metabolite spectra from various samples, the peaks in the mass spectra were integrated for all compounds, and integration correction was performed for the same substance between samples (19).

### 2.5 Data analysis

The experiment was conducted in triplicate, and the average of the three replicate peaks was used for analysis. Various statistical analysis methods were applied to the metabolite data, including T testing, principal component analysis (PCA), orthogonal partial least squares-discriminant analysis (OPLS-DA), hierarchical cluster analysis (HCA), and K-means clustering. T tests were conducted to evaluate statistical significance, and OPLS-AD and fold change (FC) were used to screen for significant differences in metabolites between treatments, with the screening standards of VIP ≥1 and FC ≥1.6 for significantly upregulated differential compounds and VIP ≥1 and FC ≤0.62 for significantly downregulated differential compounds (20). K-means clustering analysis was conducted to analyze the dynamics of metabolites between treatments, along with metabolite enrichment versus annotation analysis using the KEGG database^1^ (21), and all algorithms and mappings were implemented using the R package.

### 2.6 Chemicals

The listed chemicals were all HPLC grade. Methanol, acetonitrile, formic acid, polypropylene glycol, and DMSO were purchased from Merck Chemical Technology Co., Ltd. (Shanghai, China).

## 3 Results and discussion

### 3.1 Quality characteristics of JGY finished tea

**Figure 2:**
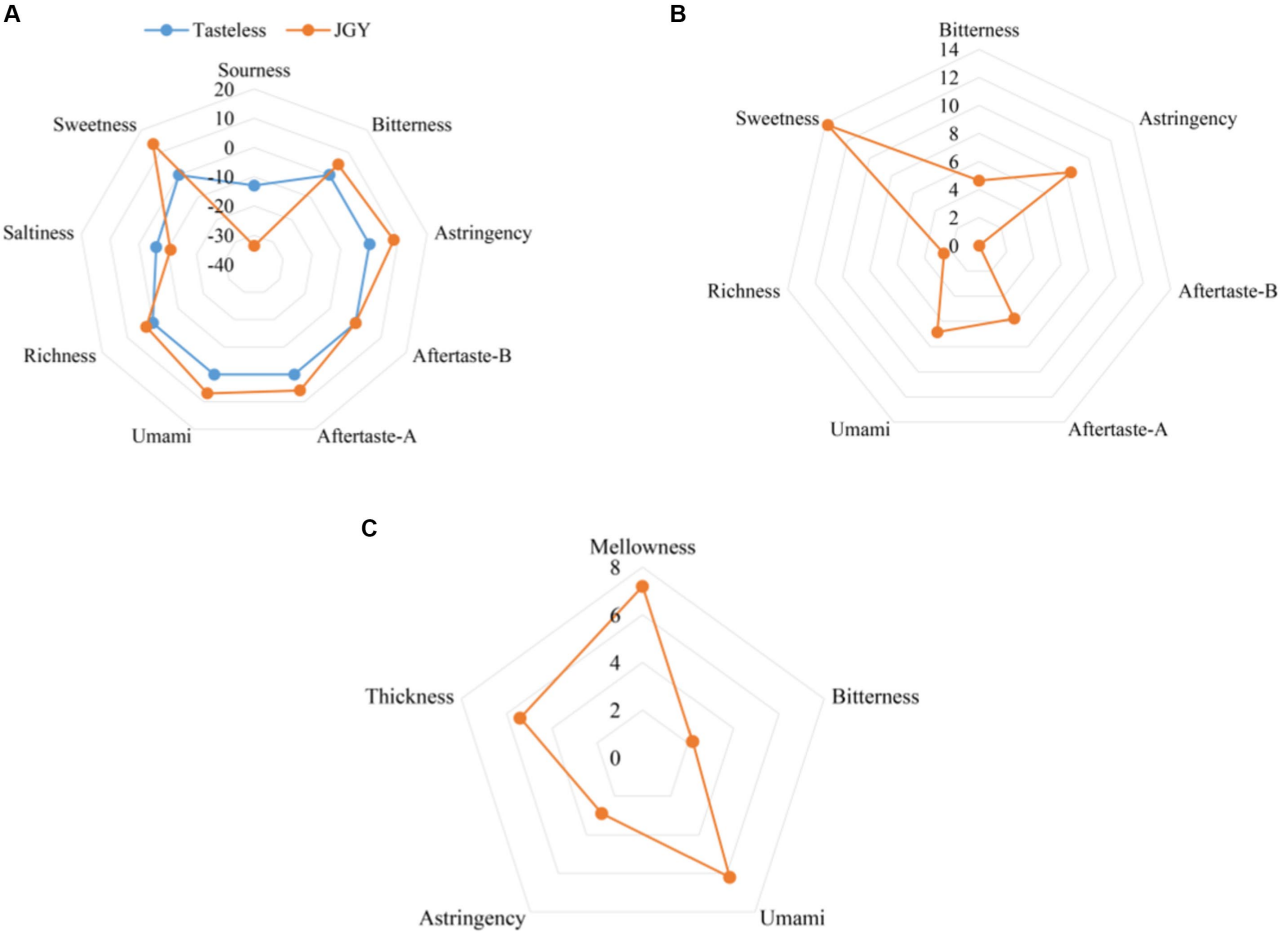
Sensory quality characteristics of JGY finished tea. **(A)** Values of various taste attributes compared with tasteless solution. **(B)** Effective values of each taste attribute. **(C)** The intensity value of quantitative description analysis by review experts.

The combination of electronic tongue (ET) and sensory evaluation make the review of tea more objective and scientific. Firstly, tasteless solution was used as the control experiment of ET (Figure 2A), sourness and saltiness were excluded owing to less than tasteless values, and other higher tasting properties were shown in Figure 2B. The difference between two points (sweetness and astringency) and tasteless values was the largest, followed by umami, aftertaste-A, and bitterness. The result of sensory evaluation was investigated in Table 2, quantitative description test was shown in Figure 2C. In summary, JGY tea products present a characteristic flavor of mellow, sweet in aftertaste, and significant Yin rhyme.

**Table 2:** The sensory evaluation of JGY tea product.

Sample ID	Appearance	Liquor color	Aroma	Taste
JGY	Tight and slender, yellowish green, smooth and clean	Golden yellow, bright	Sweet flower and fruit fragrance	Mellow, sweet in aftertaste; significant yin rhyme

### 3.2 General description of tea metabolites

**Figure 3:**
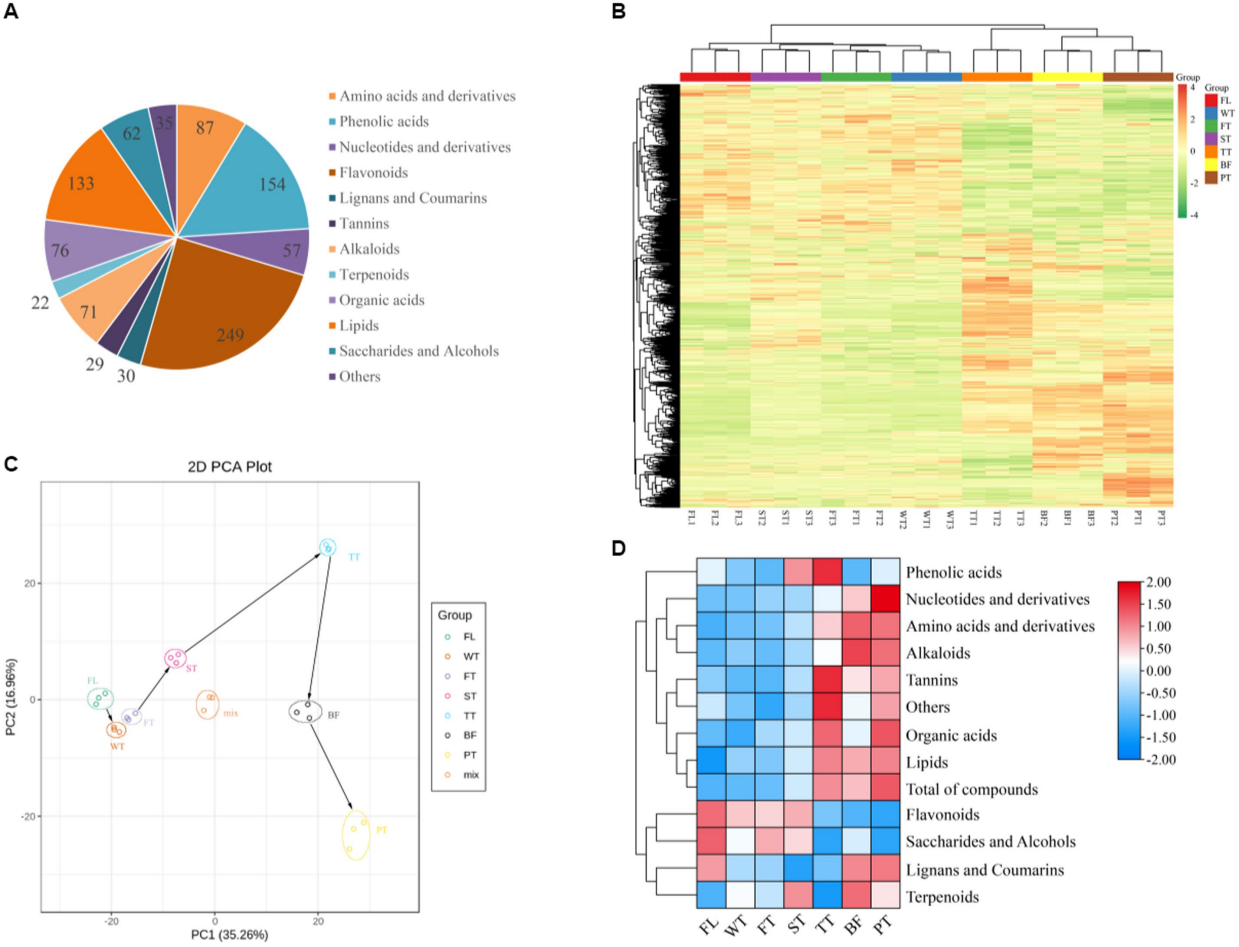
General changes in nonvolatile metabolites during oolong tea processing. **(A)** Classification of JGY detected 1,005 nonvolatile metabolites. **(B)** Cluster heatmaps of nonvolatile metabolites in samples at different processing stages. **(C)** Principal component analysis (PCA) scores of all nonvolatile metabolites. **(D)** Overall amounts of various nonvolatile metabolites during different stages of oolong tea processing.

A total of 1,005 metabolites were detected and classified into 12 major categories, including 87 amino acids and derivatives, 154 phenolic acids, 57 nucleotides and derivatives, 249 flavonoids, 30 lignans and coumarins, 29 tannins, 71 alkaloids, 133 lipids, 62 saccharides and alcohols, and 35 other components (Figure 3A). The peak areas of all metabolites and their dynamic changes during manufacturing were visualized with clustering heatmaps (Figure 3B). The results showed that FL, WT, FT and ST were clustered into one group and TT, BF and PT were clustered into another group, which indicated that some metabolites of the tea leaves presented similar trends at FL, WT, FT and ST, while others changed similarly at TT, BF and PT. Many metabolites accumulated abundantly at TT, BF and PT, suggesting that the third turning-over, third setting and high-temperature treatments could be critical elements for accumulation in some compounds. Meanwhile, the three biological replicates of each sample were aggregated together, reflecting excellent stability and reproducibility within groups.

The principal component analysis results for the tea manufacturing and quality control (mix) samples are shown in Figure 3C, with the top three principal components cumulatively contributing 60.46% (PC1 = 35.26%, PC2 = 16.96% and PC3 = 8.24%). The quality control samples (mix) were tightly clustered together and positioned in the core among all samples, indicating greater dependability and duplicability of the resulting data. The PCA plot of the manufacturing samples showed great separation between various treatment samples and changes in the metabolite fractions with the manufacturing of oolong tea. Interestingly, there was a relatively large difference between FL WT FT ST and TT BF PT in the direction of PC1, suggesting that the tea quality changes in oolong tea manufacturing were strongly related to PC1 components. This result was consistent with the results shown in the clustering heatmap.

The changes in the nonvolatile contents of diverse types in oolong tea manufacturing were calculated, as shown in Figure 3D. According to the results, the samples were arranged in terms of the total amount of nonvolatile metabolites in the following sequence: PT > TT > BF > ST > FT > WT > FL. Moreover, 9 metabolites (all studied metabolites except phenolic acid, lignans and coumarins) changed significantly during manufacturing (*p* < 0.05). Among them, the accumulation of amino acids and their derivatives and alkaloids increased constantly from FL to BF but decreased with the high-temperature treatment (PT). Phenolic acids, organic acids, tannins, and lipids achieved maximum abundance when the third turning-over ended or manufacturing was completed. Nucleotides and their derivatives displayed a sustained upward trend, while flavonoids, sugars and alcohols displayed a negative trend. Collectively, amino acids and their derivatives, alkaloids, flavonoids, sugars, organic acids and lipids underwent remarkable alterations during the manufacturing of oolong tea.

Previous research has demonstrated that the origin of amino acids during the manufacturing of oolong tea lies mainly in the degradation of proteins (22, 23); meanwhile, several soluble sugars were transformed into aromatic chemicals, such as pyrazines and pyrroles, via the Maillard reaction with amino acids under the influence of heat. The dynamic changes in amino acids and their derivatives in the current research were probably related to protein hydrolysis before the high temperature treatment and the massive occurrence of Maillard reactions between amino and carboxy compounds at high temperature. Flavonoids are key contributors to the flavor profile of tea and precursors to important metabolites, such as flavanols (catechins) and flavonoid (alcohol) glycosides (24). There was an overall consistent decreasing trend of flavonoids in oolong tea manufacturing, which indicates that flavonoids were gradually converted to other compounds, causing a decrease in their content.

### 3.3 Filtering of differential metabolites

**Figure 4:**
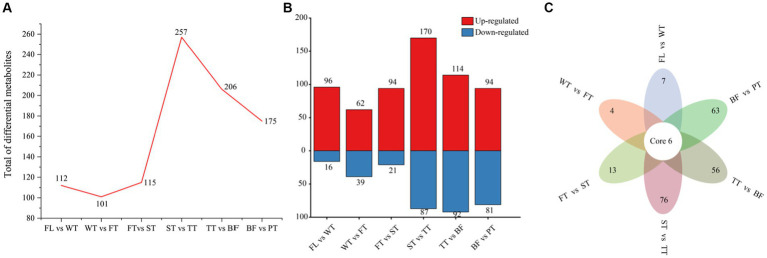
Differential metabolite profiles produced by individual processes of oolong tea manufacturing. **(A)** Differential metabolite numbers for each comparison group in oolong tea manufacturing processes. **(B)** Differential metabolite numbers with increased and decreased amounts among the comparison groups. **(C)** Venn diagram of differential metabolites for various comparison groups.

To explore the influence of the various manufacturing processes of oolong tea on diverse compounds, FL vs. WT, WT vs. FT, FT vs. ST, ST vs. TT, TT vs. BF, BF vs. PT, and FL vs. PT were compared for differential metabolite identification and analysis. The overall numbers of differential metabolites in each grouping are shown in Figure 4A, which revealed the following trend of differential metabolite numbers: TT > BF > PT > ST > WT > FT. This trend indicated that the greatest extent of metabolite variation in tea leaves was between the second turning-over and third turning-over (257), followed by TT vs. BF (206) and BF vs. PT (175), while the withered leaves (112), the first turning-over leaves (101) and the second turning-over leaves (115) were comparatively close, which was in agreement with the results of PCA and clustering heatmap analysis. Interestingly, more compounds increased in content than decreased in all groups (Figure 4B).

Common and characteristic differential metabolites in different groups were identified with Venn diagrams (Figure 4C). More specifically, six differential metabolites commonly associated with six groups (in addition to FL vs. PT) were screened, including 6-hydroxy-5,7,4′-trimethoxyflavone, 6-hydroxy-5,7,4′-trimethoxyflavone, lysoPE 16:0, lysoPC 16:2 and benzylacetone, three of which belonged to the flavonoid group and exhibited identical trends, and all reached their peak at point BF, which was possibly attributable to the oxidation of flavonoids. There were 7, 4 and 13 unique differential metabolites affected by the withering, first turning-over and second turning-over processes, respectively, while 76, 56 and 63 differential metabolites specific to the ST vs. TT, TT vs. BF and BF vs. PT groups, respectively, accounted for 89.04% of the total unique differential metabolites (219).

Hence, some compounds were absent or present in low amounts in fresh leaves of tea, but they were obtained or their contents were increased through oolong tea manufacturing (withering, the first turning-over, the second turning-over, the third turning-over, setting quietly and high-temperature treatment), and the third turning-over, setting and high-temperature treatment were the most key moments of metabolite changes in oolong tea processes.

### 3.4 Change trends of essential differential metabolites in oolong tea manufacturing

To investigate the relative content change trends of metabolites in different groups, differential metabolites that were identified according to the screening criteria in all comparative groups were subjected to z-score normalization followed by K-means clustering analysis.

**Figure 5:**
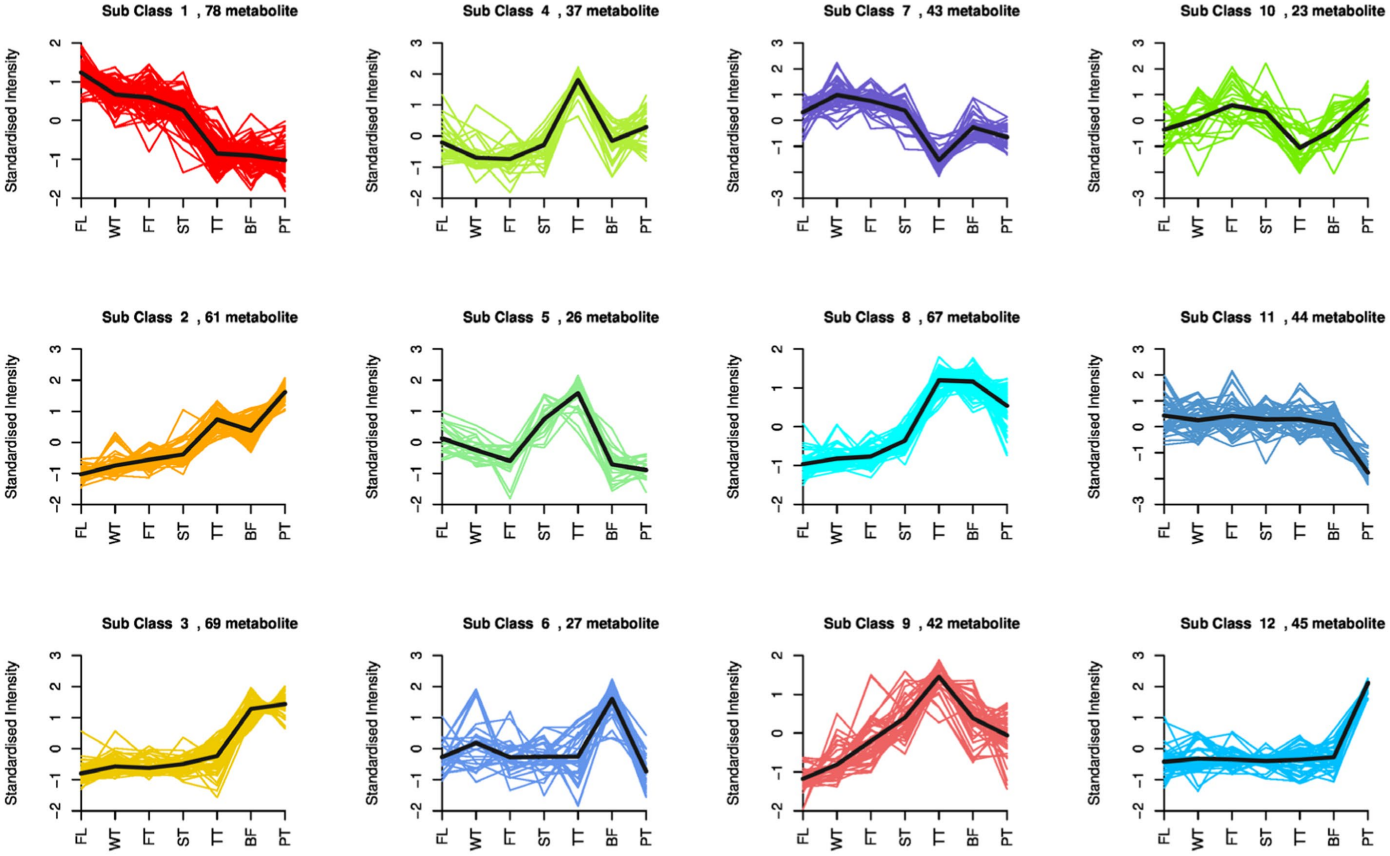
K-means clustering analysis of all differential metabolites in seven comparison groups. The *X*-axis represents the oolong tea manufacturing process, the *Y*-axis represents the standardized intensity, and different subgroups indicate different change trends in oolong tea processing.

A total of 562 differential metabolites were clustered into 12 clusters, with variation trends falling into three categories (Figure 5). Among them, the compounds of clusters 2, 3 and 12 exhibited increasing trends during oolong tea manufacturing, which mainly included indole, L-phenylalanine, L-glutamine, and dihydrokaempferol, and large accumulations of these compounds were conducive to the formation of oolong tea characteristics. Second, the compounds of clusters 1 and 11 tended to decrease in content due to being consumed, such as D-glucose, D-fructose, and 2-methoxycinnamic acid, which are important sources of secondary metabolism in oolong tea. Finally, the compounds of seven clusters (4, 5, 6, 7, 8, 9 and 10) showed fluctuating variations, with all peaking at TT or BF. In more detail, the compounds of clusters 4, 5 and 9 reached their highest peaks at TT, which consisted of γ-aminobutyric acid, jasmonic acid, xanthosine, myricetin, and citric acid; this indicates that turning over promoted the accumulation and biosynthesis of these compounds. Interestingly, compounds of clusters 11 and 12 were dramatically changed after high-temperature treatment, including naringenin, kaempferol, and naringenin chalcone, suggesting that high-temperature treatment in oolong tea manufacturing is a critical influencing factor for these compounds.

### 3.5 Dynamics of various differential metabolites in oolong tea manufacturing

A total of 562 differential metabolites were classified as shown in Supplementary Figure S1, mainly including 59 amino acids and their derivatives, 91 phenolic acids, 33 nucleotides and their derivatives, 93 flavonoids, 23 lignans and coumarins, 20 tannins, 43 alkaloids, 39 organic acids, 101 lipids, 28 saccharides and alcohols, and 18 others.

#### 3.5.1 Lipids

**Figure 6:**
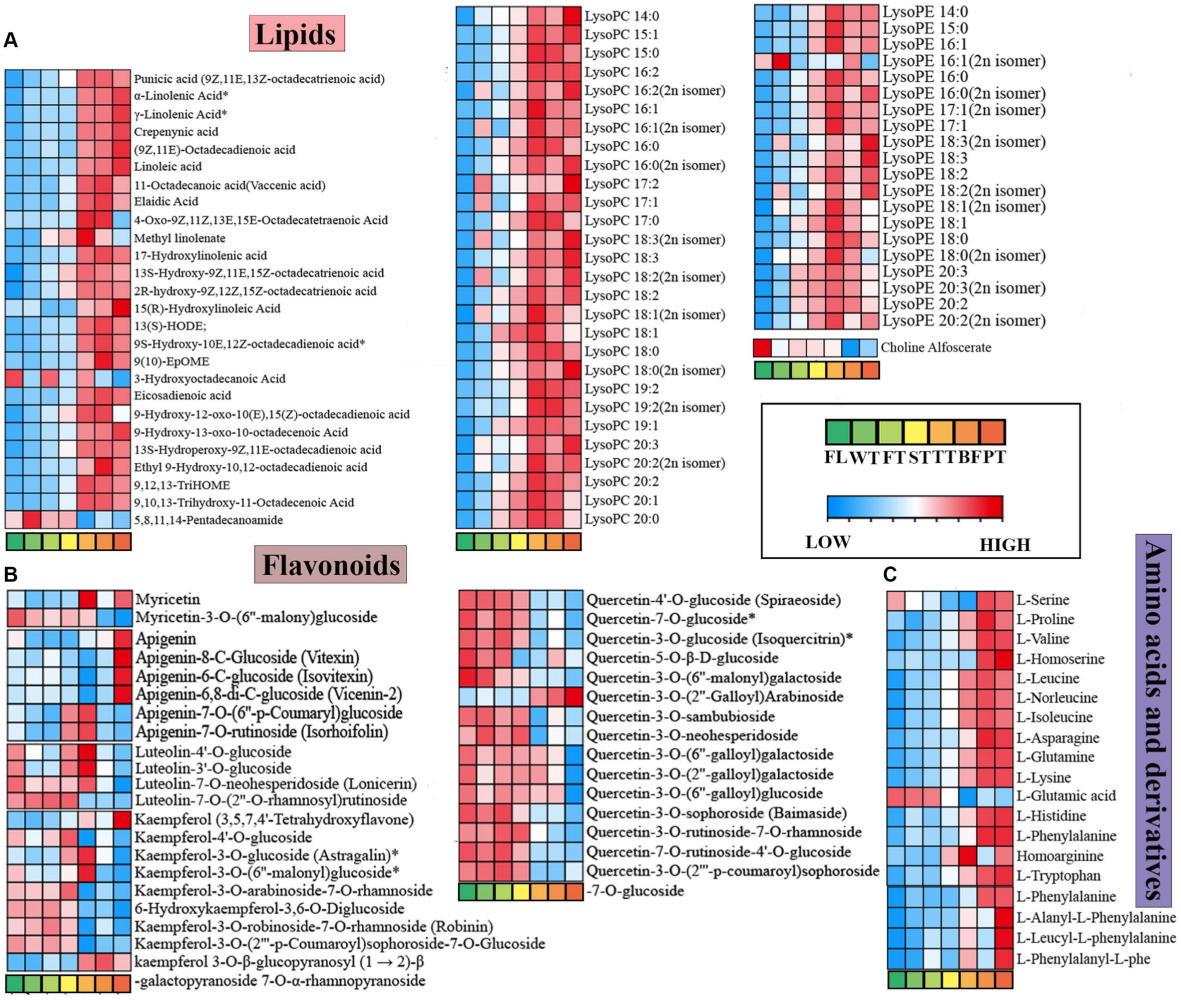
Dynamic variations of lipids, flavonoids, amino acids and derivative differential metabolites in the processing of oolong tea. **(A)** Lipids. **(B)** Flavonoids. **(C)** Amino acids and derivatives. “*” indicates a significant difference (*p* < 0.05).

Lipids are essential components of plants that are critically associated with cell membrane structure and participate in a range of physiological activities (25). Previous studies have shown that unsaturated fatty acids are important precursors to tea aroma, which yield many volatile compounds mainly through oxidation and degradation (26). In this study, 101 differential lipids were identified, which consisted of 41 free fatty acids, 28 lysophosphatidylcholines (LPCs), 20 lysophosphatidylethanolamines (LPEs), and 11 glycerol esters (Supplementary Figure S2; Figure 6A). It was found that 26 free fatty acids appeared to significantly change at TT, and 25 of them exhibited significant upward adjustments, which probably resulted from partial lipid breakdown during oolong tea manufacturing, prompting free fatty acid accumulation in large amounts. This result is consistent with the turning-over/setting process facilitating the rich floral and fruity aroma of oolong tea (27). The amounts of LPCs and LPEs increased substantially at TT. The hydrolysis products of phospholipids, including LPCs and LPEs, are essential components of cell membranes, and phospholipids have been reported to be reduced in association with alterations in lipid fractions (28). Therefore, it is speculated that the large accumulations of LPCs and LPEs were due to the turning-over process promoting massive hydrolysis of phospholipids in the tea leaves.

#### 3.5.2 Flavonoids

Flavonoids are regarded as essential colored compounds in tea soup, while compounds such as flavonoid glycosides and flavanols make overwhelming contributions to tea flavor quality. Ninety-three different flavonoid metabolites in oolong tea processing were filtered out, including 23 flavonoids, 35 flavonols, 11 dihydroflavones, and 15 flavonoid carbonosides.

Related studies revealed that the stability of flavonol glycosides was associated with aglycone and glycoside moieties (29); thus, flavonoid differential metabolites were classified by species to produce heatmaps (Figure 6B). The diagrams showed that the contents of apigenin, myricetin, kaempferol and apigenin C-glycosides (vitexin, isovitexin, and vicenin-2) increased conspicuously after high-temperature processing of oolong tea. However, the majority of 3-O-glycosides showed decreasing trends in content throughout oolong tea processing (FL vs. PT), which generally changed in the final phases of oolong tea processing (TT, BF, and PT). Previous research has shown that flavonol glycosides substituted with glycosyl groups within C-ring 3-hydroxyl groups exhibit strong antioxidant properties and that degradation can be accelerated by high temperature (30, 31), which is highly consistent with the results of this study. More exclusively, flavonol glycosides were degraded at high temperatures, which further increased the contents of myricetin, kaempferol, etc. Meanwhile, due to stronger antioxidant properties, variability occurred predominantly during the later period of oolong tea processing. Notably, apigenin C-glycosides (vitexin, isovitexin and vicenin-2) were significantly upregulated in the high-temperature treatment, which was different from the change trends of O-glycoside chemicals. This phenomenon was probably because more energy was required for C-bond formation on the apigenin C-glycosides. Consequently, the low-threshold flavonoid glycosides, which enhance the bitterness and astringency of tea broth (32), were reduced during the processing of oolong tea, suggesting that the manufacturing processes contribute to the establishment of the mellow flavor characteristics of oolong tea.

#### 3.5.3 Amino acids and derivatives

Amino acids could improve the umami and mellow taste of tea infusions, which makes them critical to the formation of tea flavor qualities (33). Fifty-nine amino acid and derivative differential metabolites were screened, including L-glutamic acid and L-phenylalanine (Figure 6C). According to the study, many amino acids presented an increasing trend followed by a decreasing trend. The switching points occurred at BF, in particular, for L-valine, L-leucine, and L-proline, as calculated from the hydrolysis of proteins. The change trend of L-glutamic acid in oolong tea processing was the opposite. As an aromatic amino acid, L-glutamic acid is an important precursor of aromatic compounds, which contributes to improving the aromatic quality of tea leaves and is oxidized to acetaldehyde during postharvest processing to produce aroma (23); thus, it was speculated that the reduction in glutamic acid content may have resulted from its conversion to aromatic compounds during fermentation and high temperature. Concurrently, as a sweet-tasting compound, the content of glutamine progressively increased with processing, correlating with that of L-glutamic acid up to −0.82. Whether a transformation relationship exists between the two amino acids in oolong tea processing merits investigation. The large accumulation of these amino acids, which correlates with the taste of the tea broth, contributes to the formation of the distinctive flavor of oolong tea (4).

#### 3.5.4 Phenolic acids

Phenolic acids are important constituents of tea polyphenols, which are closely correlated with the flavor qualities and astringency of tea leaves (34, 35). Phenolic acids exhibit extremely aggressive biological activity concerning their antioxidant properties and participate in multiple biochemical reactions during oolong tea manufacturing (36). In this paper, the maximum content of phenolic acids was attained at TT, and the content subsequently decreased under high temperature, suggesting that they were gradually generated via the metabolic pathways of shikimic acid and phenylalanine in the early period of processing and transformed into other compounds later since reactions such as redox and hydrolysis occurred.

**Figure 7:**
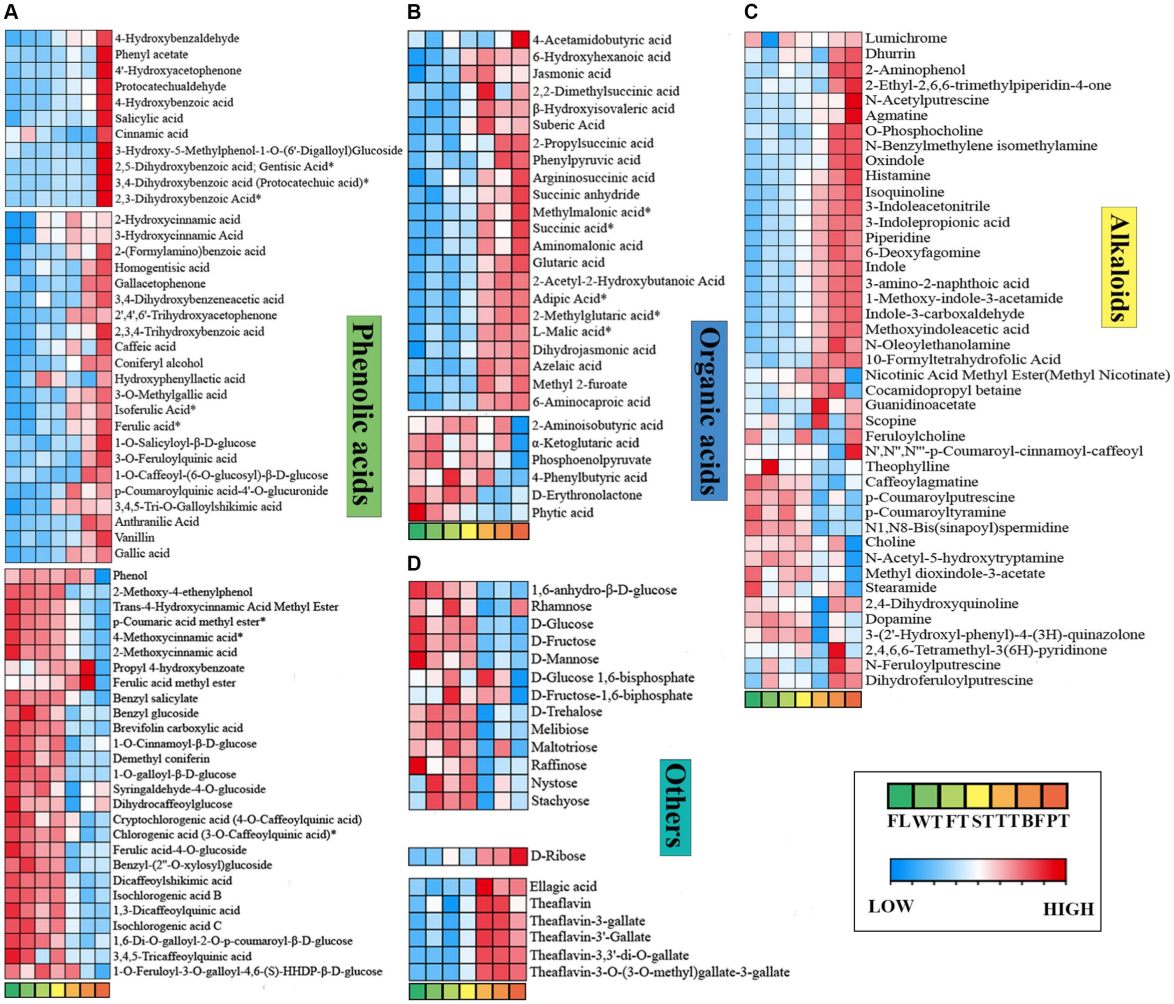
Phenolic acids, organic acids, alkaloids and other differential metabolites vary dynamically in the processing of oolong tea. **(A)** Phenolic acids. **(B)** Organic acids. **(C)** Alkaloids. **(D)** Others. “*” indicates a significant difference (*p* < 0.05).

Ninety-one phenolic acid differential metabolites were distinguished, including 33 with upward and 27 with downward significant deviations (FL vs. PT), as shown in Figure 7A. Several phenolic acids appeared to markedly accumulate after undergoing the high-temperature treatment but showed no noticeable alterations in other processes, such as salicylic acid, cinnamic acid, and gentisic acid, which are linked to the reported investigation (35), illustrating that high temperature has the greatest effect on such compounds throughout oolong tea processing. Research has shown that increases in gallic acid promote oolong tea umami formation (37). Gallic acid broadly showed an upward trend during oolong tea processing, and it decreased in BF and increased after the high temperature treatment (PT). This indicates that oolong tea processing supports umami flavor formation. Perhaps the increase in gallic acid content originates from the degradation of EGCE or its dimer at high temperatures and pretemperature tannase activity (38–40). However, the reduction is caused by oxidative polymerization of gallic acid to its derivatives at BF, such as gallic acid-4-O-glucoside and gallocatechin gallate (41).

Conversely, numerous phenolic acids, represented by p-coumaric acid methyl ester, trans-4-hydroxycinnamic acid methyl ester, and chlorogenic acid, were significantly downregulated, with p-coumaric acid methyl ester varying most drastically (FC_(FL/PT)_ = 6.39). Intriguingly, cinnamic acid was found to be massively reduced in FT, yet its downstream metabolites 2-hydroxycinnamic acid and 3-hydroxycinnamic acid increased prominently, which was probably due to cinnamic acid metabolism being facilitated by turning over. All of the above results demonstrate dramatic modifications of phenolic acids in the processing of oolong tea.

#### 3.5.5 Organic acids

Organic acids can affect the taste properties of tea as buffering agents of bitterness and astringency, which are pivotal intermediate products of carbohydrate catabolism (42–44). The total organic acid content followed an up-down-up trend and decreased in BF (Figure 7B), indicating essential physiological transformations of carbohydrates in oolong tea processing, consistent with the conclusion of the study that organic acids are related to the degree of fermentation, with heavier fermentation resulting in higher content (45). Thirty-nine different organic acids were screened, of which 22 were significantly upregulated and 6 were significantly downregulated (FL vs. PT). Among the upregulated organic acids, dihydrojasmonic acid and jasmonic acid showed noticeable variations, with their contents increasing to 40.18 and 10.73 times those in fresh leaves, respectively. More specifically, after processing oolong tea, the contents of sour-tasting L-malic acid and succinic acid increased, while that of citric acid decreased after BF. As such, organic acids in oolong tea processing present diverse variations with different types, but overall, an increasing variation contributes to the formation of tea flavor.

#### 3.5.6 Alkaloids

Alkaloids are a class of nitrogenous organic compounds, with purine alkaloids serving as principal alkaloids in tea, including caffeine, theobromine and theophylline, which play an important role in the bitterness and astringency of tea soup (46, 47). Across oolong tea processing, 43 differential alkaloids were separated, such as indole and choline (Figure 7C), while caffeine, theobromine and theophylline failed to express significant differences. Combined with the results of the metabolic pathway analysis, it was found that the contents of xanthine, 7-methylxanthine, 1,7-dimethylxanthine, theobromine, and 3-methylxanthine in the caffeine metabolism (Ko00232) pathway increased significantly after turning-over, indicating that the turning-over process is potentially beneficial to the synthesis of caffeine precursors. However, there were no significant changes in caffeine, which is in agreement with the previous conclusions that caffeine variation in oolong tea processing was not significant (48, 49).

#### 3.5.7 Other compounds

Earlier research has shown that sugar compounds provide little contribution to the sweetness intensity of tea but can serve to optimize the taste of tea broth (50). With the processing of oolong tea, the sugar compounds presented a continuous decreasing trend (Figure 7D). Moreover, 14 significantly different sugar compounds were found, and all except D-ribose were significantly downregulated, indicating that most of the sugars were consumed and converted to other compounds during the processing of oolong tea. This was presumably due to glycolysis by respiration solely after fresh leaves broke away from the tea plant (51) and to the Maillard reaction with amine compounds (52). Interestingly, the contents of sugars such as D-glucose and melibiose slightly rose after the turning-over process, probably causing turning-over to promote the degradation of polysaccharides and pectin-like compounds. Furthermore, the contents of theaflavins associated with both color and taste were highest in TT or BF, which was related to the oxidation of catechins. In brief, the processing of oolong tea reduced the sugar compound contents and increased the theaflavin contents.

## 4 Conclusion

The manufacturing processes of oolong tea are sophisticated, and assorted nonvolatile components appear to undergo extreme changes. The third turning-over, third setting and high-temperature treatment significantly affected the nonvolatile compounds of JGY during oolong tea manufacturing. The joint variations of amino acid accumulation (e.g., L-Glutamine), oxidation of flavonoids (e.g., apigenin, vitexin, and quercetin glycoside compounds), stabilization of caffeine, dynamics of phenolic acids (e.g., gallic acid) and organic acids (e.g., L-malic acid, succinic acid, and citric acid), and lipid (e.g., choline alfoscerate, LPCs, and LPEs) accumulation contributed to the formation of JGY oolong tea quality characteristics. These findings improve the understanding of the formation of unique flavor qualities during oolong tea processing.

## Data availability statement

The original contributions presented in the study are included in the article/Supplementary material, further inquiries can be directed to the corresponding authors.

## Author contributions

QH: Investigation, Software, Visualization, Writing – original draft, Writing – review & editing. YZ: Writing – original draft, Formal analysis, Software, Visualization, Writing – review & editing. YY: Methodology, Writing – review & editing. Z-XN: Investigation, Writing – original draft. BC: Methodology, Writing – review & editing. ZW: Data curation, Writing – review & editing. HH: Data curation, Writing – review & editing. QW: Formal analysis, Writing – review & editing. Z-wZ: Conceptualization, Writing – review & editing. SG: Investigation, Writing – review & editing. ZL: Resources, Validation, Writing – review & editing. HL: Resources, Writing – original draft, Writing – review & editing. YS: Funding acquisition, Resources, Writing – original draft, Writing – review & editing.

## Footnotes

^1^ https://www.kegg.jp/
