# Supplementary material for: Widely targeted metabolomics analysis reveals the formation of nonvolatile flavor qualities during oolong tea manufacturing: a case study of Jinguanyin
Source: Front Nutr. 2023 Dec 14;10:1283960. doi: 10.3389/fnut.2023.1283960 (PMC10751955; doi:10.3389/fnut.2023.1283960)
Supplement: Supplementary file 2 [file Data_Sheet_1.docx]

**Widely targeted metabolomics analysis reveals the formation of nonvolatile flavor qualities during oolong tea manufacturing: A case study of Jinguanyin**

Qingcai Hu^a,b,†^, Yucheng Zheng^c,†^, Yun Yang^a,b^, Zixin Ni^a,b^, Bin Chen^a^, Zongjie Wu^a^, Huiqing Huang^a,b^, Qingyang Wu^a,b^, Ziwei Zhou^d^, Shuilian Gao^e^, Zhongxiong Lai^b^, Hongzheng Lin^a,^* , Yun Sun^a,^*

^a^ Key Laboratory of Tea Science, College of Horticulture, Fujian Agriculture and Forestry University, Fuzhou 350002, PR China

^b^ Institute of Horticultural Biotechnology, Fujian Agriculture and Forestry University, Fuzhou 350002, PR China\

^c^College of Tea and Food Science, Wuyi University, Wuyishan 354300, China

^d^ College of Life Science, Ningde Normal University, Ningde 352100, China

^e^ Anxi College of Tea Science, Fujian Agriculture and Forestry University, Quanzhou 362000, China

^†^ These authors contributed equally to this work.

*Corresponding author at: Key Laboratory of Tea Science, College of Horticulture, Fujian Agriculture and Forestry University, Fuzhou 350002, China.

E-mail address: [331926991@qq.com](mailto:331926991@qq.com) (Hz.Lin); sunyun1125@126.com (Y.Sun).

Tel: 8615980201506 (Hz.Lin); 8613960826626 (Y.Sun).

**Supplementary Materials**


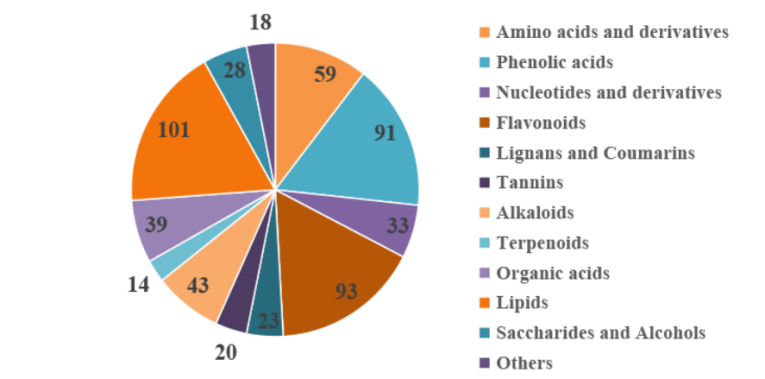


**Figure. S1** Categories of 562 differential metabolites


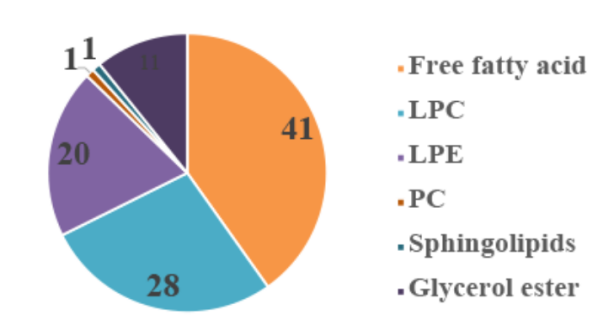


**Figure. S2** Lipid classification of differential metabolites
